# Experiences of lockdown during the Covid-19 pandemic: descriptive findings from a survey of families in the Born in Bradford study

**DOI:** 10.12688/wellcomeopenres.16317.2

**Published:** 2021-02-26

**Authors:** Josie Dickerson, Brian Kelly, Bridget Lockyer, Sally Bridges, Christopher Cartwright, Kathryn Willan, Katy Shire, Kirsty Crossley, Maria Bryant, Trevor A. Sheldon, Deborah A. Lawlor, John Wright, Rosemary R C McEachan, Kate E. Pickett, on behalf of the Bradford Institute for Health Research Covid-19 Scientific Advisory Group

**Affiliations:** 1 Bradford Institute for Health Research, Bradford Teaching Hospitals NHS Foundation Trust, Bradford Royal Infirmary, Duckworth Lane, Bradford, BD9 6RJ, UK; 2 Hull York Medical School, University of York, Heslington, York, YO10 5DD, UK; 3 Department of Health Sciences, University of York, Seebohm Rowntree Building, Heslington, York, YO10 5DD, UK; 4 Institute of Population Health Sciences, Barts and The London School of Medicine and Dentistry, Queen Mary, University of London, Yvonne Carter Building, 58 Turner Street, London, E1 2AB, UK; 5 MRC Integrative Epidemiology Unit, University of Bristol, Oakfield House, Oakfield Grove, Bristol, BS8 2BN, UK; 6 Population Health Science, Bristol Medical School, University of Bristol, Bristol, BS8 2BN, UK; 7 Bristol National Institute for Health Research Biomedical Research Centre, University of Bristol, Bristol, BS8 2BN, UK

**Keywords:** Covid-19, mental health, poverty, health inequalities, ethnicity, social determinants of health, cohorts, Born in Bradford

## Abstract

**Background**: Lockdown measures implemented to contain the Covid-19 virus have increased health inequalities, with families from deprived and ethnically diverse backgrounds most likely to be adversely affected. This paper describes the experiences of families living in the multi-ethnic and deprived city of Bradford, England.

**Methods**: A wave of survey data collection using a combination of email, text and phone with postal follow-up during the first Covid-19 UK lockdown (10th April to 30 ^th^ June 2020) with parents participating in two longitudinal studies. Cross tabulations explored variation by ethnicity and financial insecurity. Text from open questions was analysed using thematic analysis.

**Results**: Of 7,652 families invited, 2,144 (28%) participated. The results presented are based on the 2,043 (95%) mothers’ responses: 957 (47%) of whom were of Pakistani heritage, 715 (35%) White British and 356 (18%) other ethnicity 971 (46%) lived in the most deprived decile of material deprivation in England. and 738 (37%) were financially insecure.

Many families lived in poor quality (N=574, 28%), overcrowded (N=364, 19%) housing. Food (N=396, 20%), employment (N=728, 37%) and housing (N=204, 10%) insecurities were common, particularly in those who were furloughed, self-employed not working or unemployed. Clinically important depression and anxiety were reported by 372 (19%) and 318 (16%) mothers. Ethnic minority and financially insecure families had a worse experience during the lockdown across all domains, with the exception of mental health which appeared worse in White British mothers.  Open text responses corroborated these findings and highlighted high levels of anxiety and fear about Covid-19.

**Conclusions**: There is a need for policy makers and commissioners to better support vulnerable families during and after the pandemic. Future work will use longitudinal data from before the pandemic, and from future surveys during the pandemic, to describe trajectories and the long-term consequences of the pandemic on vulnerable populations.

## Introduction

In response to the Covid-19 pandemic, the UK government, like many others internationally, has implemented stringent lockdowns to stop the spread of the virus ^1^. In the first UK lockdown, implemented from March 23 ^rd^ 2020, this included the closure of all schools, non-essential shops and businesses, reduced health and social care provision and restrictions on daily activities, with the aim of limiting the number of deaths, severe Covid-19 cases and consequent pressures on the National Health Service (NHS) ^2^. There is a growing recognition that the measures have had a negative impact on mental health ^3, 4^ and economic insecurity ^5, 6^, with the greatest impact most likely to be on those in society who are already vulnerable ^7– 9^.

The Born in Bradford (BiB) research programme has been following the health and wellbeing of over 36,000 Bradford residents since 2007. Bradford is the fifth largest metropolitan district in England, situated in the North of England and has a young, ethnically diverse population with high levels of deprivation and health inequalities ^10^. BiB is host to three family cohort studies, two of which had collected in-depth information on the demographics of participants prior to the pandemic, as well as consent to contact participants for new research studies ^11– 13^. We were therefore in a unique position to be able to study the impact of the Covid-19 pandemic and lockdown response on families with pre-school, primary and/or secondary school aged children living in a highly deprived and ethnically diverse city.

This research infrastructure is being harnessed as part of a mixed-methods, longitudinal adaptive research study to provide actionable intelligence to local decision makers about how best to minimise health inequalities and aid the City’s recovery. This includes plans to use longitudinal data from before the pandemic, and from future surveys during the pandemic, to describe the trajectories, and identify the long-term consequences, of the pandemic on vulnerable populations. Our approach can be read in more detail here ^10^.

This paper reports the participant characteristics and the immediate health, social and economic status, by ethnicity and financial insecurity, during the first survey of BiB parents undertaken during April – June 2020.

The main objectives of this initial analysis were:

• To explore differential impacts of the first UK Covid-19 lockdown, by ethnicity and current financial insecurity, on living circumstances, including food, employment and housing insecurity, and the physical and mental health of parents.

• To explore responses to open-ended questions about worries, challenges and any positive experiences during lockdown, in order to corroborate the survey findings and identify any other areas of concern.

• To inform areas of interest, including health and socioeconomic issues, for repeated longitudinal waves of data collection throughout the pandemic.

• To identify research questions for further in-depth quantitative analysis using pre-Covid-19 baseline data from these longitudinal studies.

## Methods

### Study design

Adult participants from two prospective birth cohort studies were asked to complete a survey of their experiences during the first full lockdown response to the Covid-19 pandemic, which was implemented from 23 March 2020.

### Study population

1. Born in Bradford Growing Up (BiBGU) (N= 5,154, 2017–2020). Sample: Parents with an index child aged 9–13 ^11, 12^.

2. Born in Bradford’s Better Start (BiBBS) (N=2,665, 2016–2019). Sample: Parents with an index child aged 0–4 ^13^.

### Mode of delivery and data collection

We used multiple methods - a combination of emails, text and phone with a follow-up postal survey in order to facilitate a rapid response. Participants were recruited in their main language wherever possible.

### Consent

Participants had previously consented to be a part of Born in Bradford and for their research and routine health and education data to be used for research. For this survey, and as approved by the HRA and Bradford/Leeds research ethics committee, verbal consent was taken for questionnaires completed over the phone and logged in the questionnaire database, implied consent was assumed for all questionnaires completed via post or online.

### Measures

Key questionnaire domains for the survey were co-produced with the Bradford Institute for Health Research Covid-19 Scientific Advisory Group ^14^ and key policy and decision makers within Bradford, based upon hypothesised areas of impact for vulnerable families and their likely mediators. Questions were selected from validated questionnaires, from previous Born in Bradford questionnaires or were devised specifically for this survey. The full questionnaire is available on our website ^14^ and as *Extended data* ^15^, key domains were:

• Household Circumstances: number of children, adults and bedrooms in the house; housing tenure ^16^, quality of housing; clinical vulnerability to, and self-isolation due to Covid-19 ^17^.

• Family relationships and social support: partner relationship ^18^, parenting competency ^19^, social support, and loneliness ^20^.

• Financial security; employment security ^21^; housing security; food insecurity ^22^.

• Physical Health ^23^ and health anxiety ^24^; smoking, alcohol and physical activity.

• Mental Health: depression (PHQ-8 ^25^) and anxiety (GAD-7 ^26^).

• Main worries, challenges and any positive aspects of lockdown: open ended questions.

Ethnicity was captured in self-reported questionnaires administered at baseline recruitment to the cohorts (March 2007 to December 2010 for BiB; January 2016 to February 2020 for BiBBS); and categorised as ‘White British’, ‘Pakistani Heritage’ and Other (there were small numbers of non-White British, non-Pakistani Heritage parents from multiple ethnic groups). We linked residential address (as at 31st March 2019) to the 2019 Index of Multiple Deprivation ^27^.

### Statistical analysis

Descriptive statistics are presented for each of the survey domains. We used cross tabulations (proportions and 95% confidence intervals) to explore differences in outcomes (e.g. forms of economic insecurity and mental health outcomes) by ethnicity and current financial security to look for different experiences based on these key vulnerabilities. For financial insecurity we used the question: “How well would you say you are managing financially right now?”. Answer options are: living comfortably, doing alright, just about getting by, finding it quite difficult, finding it very difficult. The latter two options were grouped and categorised as indicating current financial insecurity.

For depression we used total scores on the PHQ8 and standard categorisations (0 to 4 no depression, 5 to 9 mild depression, 10 to 14 moderate depression, 15 to 19 moderately severe depression and 20 to 24 severe depression) ^25^. Similarly, for anxiety we employed total scores on the GAD7 and standard categorisations (0 to 4 no anxiety, 5 to 9 mild anxiety, 10 to 14 moderate anxiety and 15 to 21 severe anxiety ^26^). Moderate, moderately severe and severe categories were collapsed to indicate clinically important symptoms of depression and anxiety.

The majority of respondents (95%) were mothers, with the remaining 5% being partners. As most partners lived in the same households as the mothers, we completed analyses using the mothers’ response to avoid duplicate responses on household questions. Cross tabulations of partners responses were completed to look for differences in outcomes of partners compared to mothers. All statistical analyses were carried out using Stata 15 ^28^.

Text responses to the open questions were explored by thematic analysis ^29^. The first 100 responses were analysed by one researcher (BL), employing an inductive approach where coding and theme development were driven by the content of the responses. Two codebooks were developed, one for the questions on the three biggest worries and recent challenges during lockdown and another smaller codebook for the question on what had been made more enjoyable and easier during lockdown. The remaining responses were then coded by three different researchers in order to test the strength and validity of the codebooks. Through frequent discussion between the researchers about this process, adjustments were made to the original codebooks so that they were reflective of the total responses.

### Ethics

This research was approved by the HRA and Bradford/Leeds research ethics committee (BiB Growing Up study 16/YH/0320; BiBBS study 15/YH/0455).

## Results

**Figure 1.**
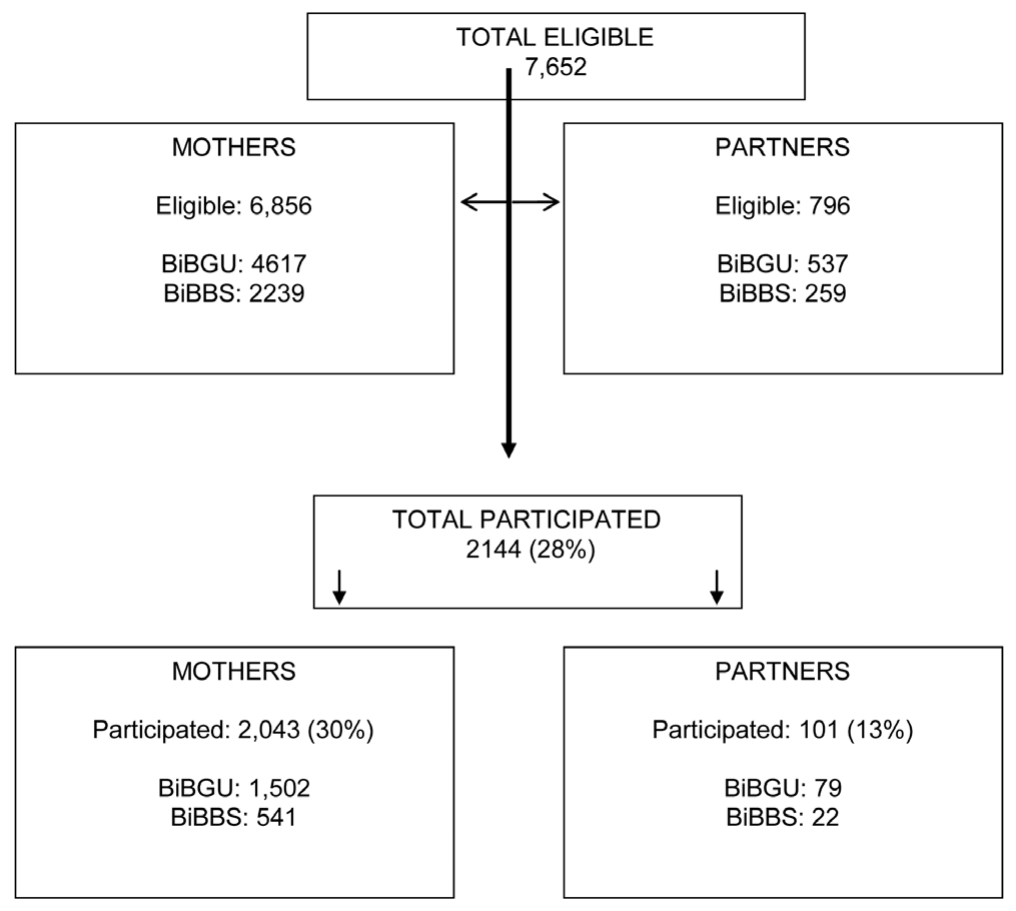
CONSORT diagram.

Out of a total of 7,652 eligible participants, 2,144 (28%) participated in this first study between 10th April and 30 ^th^ June 2020. 2,043 were mothers and 101 partners, of whom 64 were from the same household as a mother in the sample. 1,581 (74%) were from the BiBGU cohort and 563 (26%) from the BiBBS cohort (see Figure 1).

Participants had a mean age of 38 years (SD 7); the mean age in the BiBGU cohort was 40 (SD 6) and in the BiBBS cohort it was 32 (SD 6). 957 (47%) are of Pakistani heritage, 715 (35%) of White British ethnicity and 356 (18%) of other ethnicities. A total of 971 (46%) respondents live in the most deprived decile of material deprivation in England (IMD 2019). The participants were broadly representative of the BiB and BiBBS cohorts (see Table 1).

**Table 1.** Characteristics of the sample and comparison to the birth cohorts.

	BiB Cohort	BiBGU Cohort	BiBBS	BiBGU Covid Sample (N=1581)	BiBBS Covid Sample (N=563)	Total Covid Sample (N=2144)
**Ethnicity**						
White British	38%	28%	13%	638 (44%)	68 (13%)	706 (36%)
Pakistani Heritage	45%	56%	64%	600 (41%)	322 (63%)	922 (47%)
Other	17%	15%	23%	222 (15%)	123 (24%)	345 (17%)
Missing				121	50	171
Age *						
Mean (S.D.) in years	39 (6)	39 (6)	31 (6)	40 (6)	32 (6)	38 (7)

*Age as at 1 ^st^ April 2020 for baseline cohort and date of survey for Covid sample (April to June 2020).

BiB, Born in Bradford; BiBGU, Born in Bradford Growing Up; BiBBS, Born in Bradford’s Better Start.

### Financial insecurity

When asked about current financial security, 403 (20%) of families were living comfortably, 857 (43%) were doing alright, 501 (25%) were just about getting by and 237 (12%) were finding it difficult or very difficult to manage. 627 (33%) respondents said they were worse off during lockdown compared to three months previously and 88 (9%) were better off. White British families were more financially secure, and more often reported being better off during lockdown. Families of Pakistani heritage were the least financially secure and more often reported being worse off during the lockdown (see Table 2).

**Table 2.** Current Financial Insecurity by ethnicity.

	White British (N=706)		Pakistani heritage (N=922)		Other ethnicity (N=345)		All (N=2,043)	
	N	Percentage (95% CI)	N	Percentage (95% CI)	N	Percentage (95% CI)	N	Percentage (95% CI)
**Current financial status**								
Living comfortably	188	27% (24%-30%)	149	17% (14%-19%)	53	16% (13%-21%)	403	20% (18%-22%)
Doing alright	311	44% (41%-48%)	358	40% (37%-43%)	158	48% (43%-54%)	857	43% (41%-45%)
Just about getting by	156	22% (19%-25%)	246	27% (25%-30%)	85	26% (21%-31%)	501	25% (23%-27%)
Finding it quite/ very difficult	49	7% (5%-9%)	146	16% (14%-19%)	32	10% (7%-13%)	237	12% (11%-13%)
Missing	2		23		17		45	
Total	706	100%	922	100%	345	100%	2043	100%
**Change in financial status compared to 3 months ago**								
Better off	88	13% (10%-15%)	58	7% (5%-9%)	29	9% (7%-13%)	179	9% (8%-11%)
About the same	421	61% (57%-65%)	471	56% (53%-59%)	176	56% (51%-62%)	1100	58% (55%-60%)
Worse off	181	26% (23%-30%)	310	37% (34%-40%)	107	34% (29%-40%)	627	33% (31%-35%)
Missing	16		83		33		137	
Total	706	100%	922	100%	345	100%	2043	100%

Those families that were financially insecure were more likely to report being worse off now than before lockdown. In contrast, those that were living comfortably were more likely to report being better off during lockdown (Supplementary Table 1, *extended data*).

**Figure 2.**
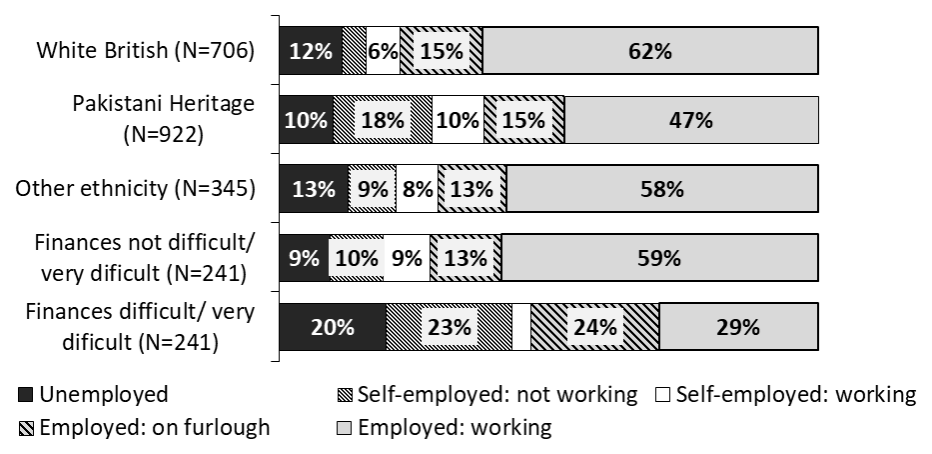
Employment status by financial insecurity and ethnicity.

The first UK lockdown had a large impact on current employment status with 228 (11%) of main earners within the household currently self-employed but not working and 292 (15%) furloughed ^1^. Table 3 shows that White British families were more likely to be still working and that Pakistani families were more likely to be self-employed but unable to work. Financial insecurity was associated with households where the main earner was self-employed and not working, furloughed, or unemployed (see Figure 2). The most secure households were those where the main earner was employed and still working. It is plausible that the financial insecurities identified here may be explained by the type of employment held by the main earner in the household, rather than being directly related to ethnicity.

**Table 3.** Current employment status by financial security and ethnicity.

	Employed (N=1,085)		Employed on furlough (N=292)		Self-employed and working (N=163)		Self-employed, not working (N=228)		Unemployed (N=221)		All (N=2,043)	
	N	Percentage (95% CI)	N	Percentage (95% CI)	N	Percentage (95% CI)	N	Percentage (95% CI)	N	Percentage (95% CI)	N	Percentage (95% CI)
**Ethnicity**												
White British	436	62% (59%-66%)	106	15% (13%-18%)	44	6% (5%-8%)	30	4% (3%-6%)	82	12% (10%-14%)	706	100%
Pakistani	426	48% (44%-51%)	132	15% (13%-17%)	84	9% (8%-11%)	163	18% (16%-21%)	89	10% (8%-12%)	922	100%
Other	191	58% (53%-63%)	41	13% (9%-17%)	25	8% (5%-11%)	29	9% (6%-12%)	42	13% (10%-17%)	345	100%
Missing	32		13		10		6		8		70	
Total	1085	55% (52%-57%)	292	15% (13%-16%)	163	8% (7%-9%)	228	11% (10%-13%)	221	11% (10%-13%)	2043	100%
**Current financial status**												
Living comfortably	293	27% (25%-30%)	42	15% (11%-19%)	36	23% (17%-30%)	11	5% (3%-9%)	14	7% (4%-11%)	403	20% (18%-22%)
Doing alright	513	48% (45%-51%)	106	37% (31%-43%)	76	48% (40%-56%)	68	31% (25%-37%)	81	38% (31%-44%)	857	43% (41%-45%)
Just about getting by	201	19% (17%-21%)	85	30% (25%-35%)	39	25% (18%-32%)	89	40% (34%-47%)	74	34% (28%-41%)	501	25% (23%-27%)
Finding it quite/ very difficult	65	6% (5%-8%)	55	19% (15%-24%)	8	5% (3%-10%)	54	24% (19%-30%)	46	21% (16%-27%)	237	12% (11%-13%)
Missing	13		4		4		6		6		45	
Total	1085	100%	292	100%	163	100%	228	100%	221	100%	2043	100

### Food, employment and housing insecurity

A high number of participants reported insecurities in food, employment and housing (see Supplementary Tables 2a-c, e *xtended data*).

Food insecurity was frequently reported with 396 (20%) mothers saying that their food often didn’t last and they couldn’t afford to buy more, and 180 (9%) having to regularly cut the size of, or skip, meals because there wasn’t enough money for food. 204 (10%) of households were worried about losing their home (eviction/repossession) and 728 (37%) families were worried about the job security of the main earner.

**Figure 3.**
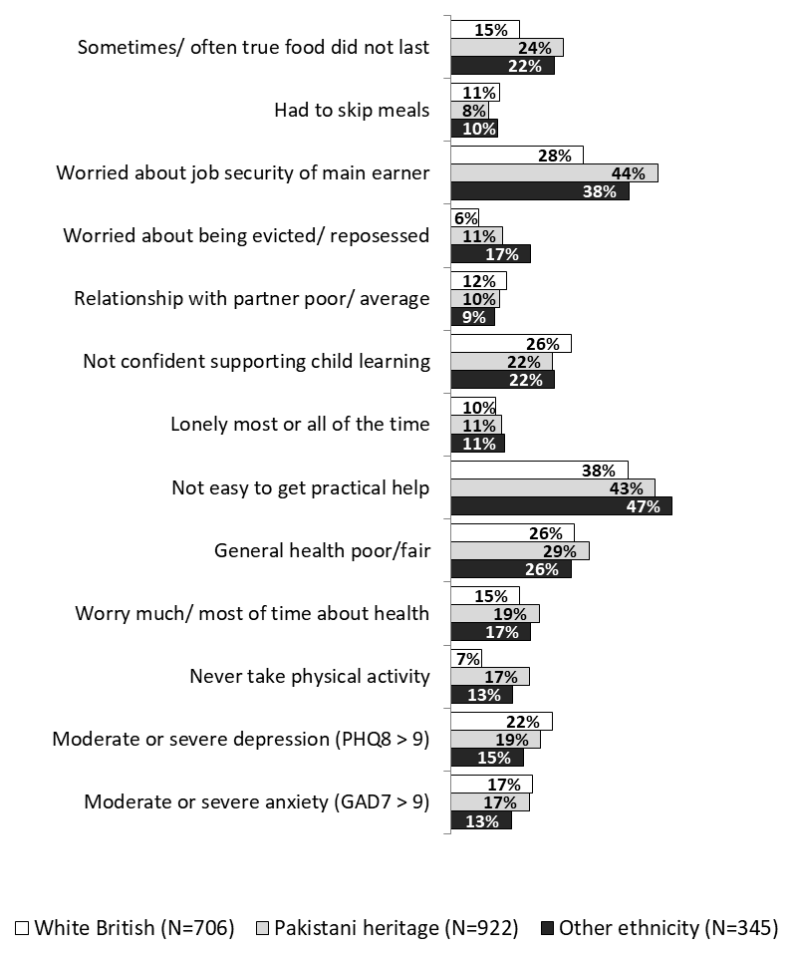
Ethnic differences across key domains.

**Figure 4.**
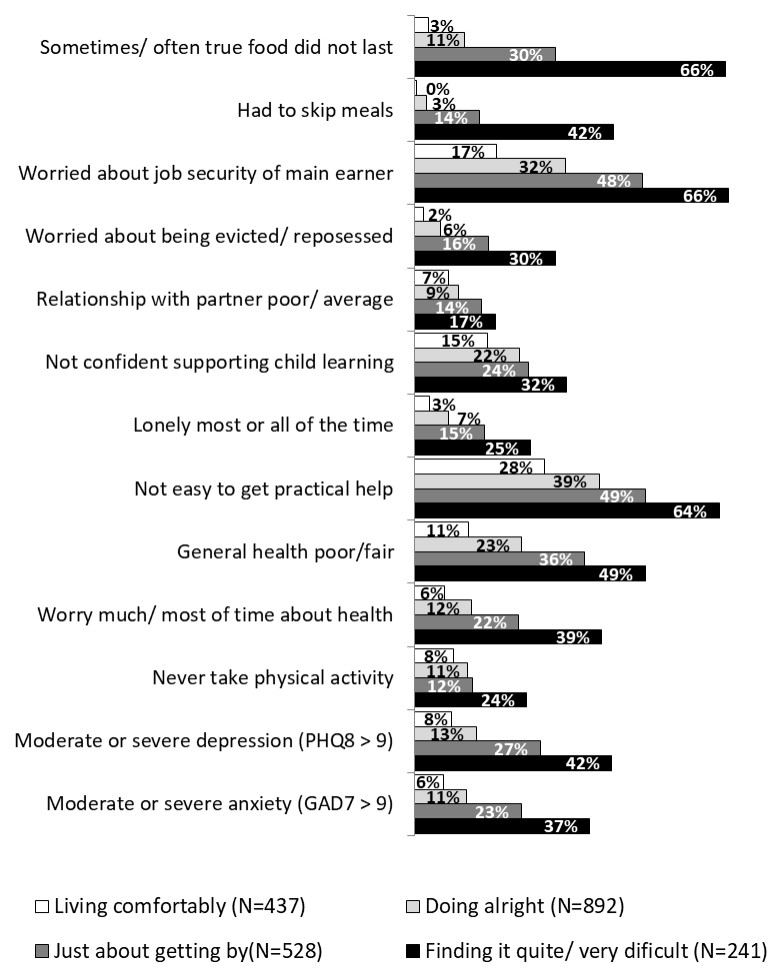
Financial insecurity differences across key domains.

Figure 3 shows that White British families were more food, employment and housing secure whilst families of Pakistani heritage were the least secure in terms of food and employment and the ‘other’ ethnic group was the least housing secure. Figure 4 shows that financial insecurity was strongly associated with food, employment and housing insecurity, whereas financial security appeared to protect against food, employment and housing insecurity.

### Household circumstances

A large proportion of families live in poor quality and overcrowded housing with damp/mould (N=574, 28%), vermin problems (N=347, 17%), major repairs needed (N= 262, 13%) and 364 (19%) reporting two or more people per bedroom. Poor quality housing was most common in families of Pakistani heritage and in those who were financially insecure. Overcrowding was most common in families of Pakistani heritage and other ethnic groups compared to White British families; it was also more common in those who were financially insecure. 464 (23%) lived in a household with someone clinically vulnerable to Covid-19 (advised to shield, >70 or pregnant), this was more common in Pakistani heritage families than White British and other ethnic groups, but there were no differences based on financial security. 558 (28%) reported that their household had self-isolated at some point, there were no ethnic differences in self isolation, however, those that were financially insecure were more likely to have needed to self isolate (see Figure 3 & Figure 4 and Supplementary Table 3, *extended data*).

### Family relationships

A large majority of mothers (N=1,770, 87%) were married or in a relationship. Most mothers reported an excellent or good relationship with their partner (N=1,537 90%); 180 (10%) reported their relationship as average/poor. There was no ethnic difference in relationship quality ( Figure 3), however an average/poor relationship was more likely in those who were financially insecure ( Figure 4). Other variables associated with an average/poor relationship included being, in poor health and those with moderate/severe depression or moderate/severe anxiety (see Supplementary table 4, e *xtended data*).

A lack of confidence in supporting home learning was reported by 414 (24%) mothers, there were no ethnic differences ( Figure 3), but a lack of confidence was more common in those with severe financial insecurity ( Figure 4). Other variables associated with a lack of confidence included poor health, moderate-severe depression and moderate/severe anxiety. (see also Supplementary table 5, *extended data*).

### Social support and isolation

607 (31%) mothers reported feeling lonely some of the time, and 199 (10%) reported feeling lonely most or all of the time. White British mothers were more likely to feel lonely some of the time, but there were no ethnic differences in those feeling lonely most/all of the time ( Figure 3). Loneliness (most/all of the time) was more common in those who were financially insecure ( Figure 4). Other variables associated with loneliness were unemployment, poor health, moderate/severe depression or moderate/severe anxiety (see Supplementary table 6, e *xtended data*).

A minority of participants (N=264, 13%) said it was difficult to get practical help from friends, family or neighbours, if they needed it. This was most common in mothers from other ethnic groups ( Figure 3), and in those who were financially insecure ( Figure 4). Other associated variables were unemployment poor health, and those with moderate-severe depression and moderate-severe anxiety (Supplementary table 7, *extended data*).

### Physical health and health behaviours

A low percentage of mothers reported having poor general health (N=135, 7%). There were no ethnic differences ( Figure 3), but this number more than doubled in households that were financially insecure ( Figure 4). Other associations were with unemployment, and poor housing conditions (see Supplementary table 8, e *xtended data*).

A large proportion of mothers who smoked (N=191, 9%) or drank alcohol (N=533, 26%) reported smoking more (N=70, 37%) and drinking more (N=164, 31%) during the first lockdown. Nearly half of mothers (N=832, 47%) reported doing less physical activity in lockdown than before. 118 (6%) reported that their children did no physical activity at all, 300 (15%) did exercise 1–2 times a week. Mothers of Pakistani heritage were most likely to do no physical activity ( Figure 3) as were those who were financially insecure ( Figure 4). Other associated variables were poor health, moderate/severe depression or moderate/severe anxiety (see Supplementary table 9, e *xtended data*).

341 (17%) of mothers reported worrying about their health most or all of the time. There were no ethnic differences in those who worried most or all of the time ( Figure 3), however this was more common in those who were financially insecure ( Figure 4). Other variables associated were unemployment, poor quality housing, and where someone in the home was clinically vulnerable to Covid-19 and/or if the household had self-isolated at some point (Supplementary table 10, e *xtended data*).

### Mental health

Reports of depression and anxiety symptoms were high with 838 (43%) mothers reporting depression, 19% of whom had clinically important (moderate/severe) symptoms. 762 (39%) mothers reported anxiety, 16% of whom had clinically important (moderate/severe) symptoms. White British participants were most likely to be moderate/severely depressed but there were no ethnic differences in moderate/severe anxiety ( Figure 3). Moderate/Severe depression and moderate/severe anxiety were associated with financial insecurity ( Figure 4). Other associations were with food, employment and housing insecurity, unemployment, poor quality housing, having self-isolated at some point, poor physical health, loneliness and a lack of social support (see Supplementary Table 11, e *xtended data)*,

### The experiences of partners

101 (5% of all responses) partners completed the first survey making it difficult to generalize from their responses. Partners who responded were more likely to be White British (N= 38, 58%) and were more financially secure (N=34, 34% were living comfortably) than the mothers overall, but showed little difference to the mother’s responses for all other measures, see Supplementary Table 12 ( *Extended data*) ^29^.

### The lived experiences of participants

We asked participants to tell us their biggest three worries at the time, a challenge they had faced in the last two weeks, and whether anything had been made easier or more enjoyable in lockdown. Some of the most commonly reported worries reflected those captured in the survey including financial insecurity and mental health concerns; other reported worries reflected concerns and challenges specific to Covid-19 including health anxieties, caring for or educating their children at home, seeing and supporting their family and friends, bereavement and wider fears about the impact of Covid-19 on society.

***Health anxieties.*** The most commonly reported worry was that they themselves, their children and/or wider family members might catch the coronavirus and become seriously ill or die. Participants were particularly concerned if they perceived themselves or others to be at a heightened risk due to existing health conditions like diabetes and asthma or if someone in the household was a key worker:

*‘I worry about contracting coronavirus particularly whilst at work and either becoming critically myself unwell or bringing it home to my family’*

*‘[I have] underlying health conditions so worried about becoming susceptible to the virus and how body would cope’*

These health anxieties had changed people’s behaviour with some reporting being too scared to go out at all or infrequently:

*‘I worry that I might die if I catch coronavirus and I worry how this will affect my children. I’m terrified they will be separated as I have 2 children with my ex-husband and one with my current. So I haven’t been outside in 10 weeks’*

*‘I'm worried my children are at risk if we go out so I only go out once a week at the weekend for a walk’*

Respondents also often reported worrying about not seeing wider family and friends and being unable to help loved ones, especially if they lived far away or in another country.

*‘Not being able to physically be there for my parents, dad very elderly and vulnerable and worrying if they died in these circumstances not being able to see them’*

Some were experiencing recent bereavement of friends or family members, both Covid-19 and non-Covid 19 deaths, and were sad and distressed about being unable to attend their funerals and gather with loved ones for support.

*‘I lost my mother 6 weeks ago and have not really been with it in terms of keeping up with my children's school work. Not really got grieve as people normally do’*

*‘Uncle passed away of Covid 19 - made it very real, can happen to anyone, happened so fast - couldn't be there with him, couldn't go to funeral. Did Boris, prince Charles get better care than him?’*

***Financial insecurity.*** The second most commonly reported worry was around financial, housing and employment insecurity. Some respondents reported that either they and/or their partner had already lost their job as a direct result of coronavirus and the lockdown, and many more were worried about this eventuality. It was clear, even from these brief responses, that for many participants, any change to income was a serious concern because they were only just managing to get by before the pandemic. For families already reliant on debt (credit cards and overdrafts) to get by, Covid-19 and lockdown measures had pushed them into deeper and longer-term debt.

*‘I have maxed my credit card .. so I'm worrying how im going to pay it back and when I'm going to be able to pay it back’*

*‘We are surviving only on child credit tax and unable to pay rent, insurance even council tax all accounts are on overdraft limit’*

Some reported having to work despite being advised not to due to Covid-19 vulnerability, or being subject to poor treatment from employers.

*‘Husband's job, it took ages for his boss to furlough him and he's threatened him that he wont have a job when it ends. He also makes him come into work some days as he does with other staff who are furloughed’*

*I was advised to self-isolate with my husband as he is classed a vulnerable person. I could not do this as I would not of had the money to pay bills or buy food’*

***Mental health.*** Respondents frequently reported concerns about their mental health, such as existing mental ill health getting worse, and the mental load of managing work, home-schooling, childcare and domestic tasks alongside wider anxiety about their family’s health, increased money worries and concerns about what the future held.

*‘Finding working from home and looking after children very demanding. No time alone. No silence. Surrounded by people and electronics all my waking hours.’*

*‘Balancing all our responsibilities - home schooling / going into work / working from home / housework - Feeling stressed’*

*‘‘I suffer from anxiety and because the situation we are in I feel like my anxiety has increased as I can’t do the normal things which I would normally do’*

Some respondents reported not being able to access or feeling unable to access mental health services due to Covid-19 and lockdown measures:

*‘Not getting my mental health support since the lockdown. My cpn. not returning your calls. It has made me a lot worse. I try to talk to my husband so i am not keeping everything inside’*

*‘Mental health, I have had previous issues in the past and am struggling and don't feel like I can approach my GP at the minute as it isn't an emergency’*

Parents also frequently reported concerns about their children’s mental health, particularly older children aged 11–16. They were considered to be particularly affected by lockdown and not being able to attend school as this was a time being able to socialise outside the family was more important. In addition, participants with children with special educational needs were especially concerned about their mental health and wellbeing, feeling less able to access sources of external support and worried about the long-term effects:

*‘I worry about my eldest child's mental well-being as she hates not being able to socialise. She does not like playing outside alone’*

*‘My 13 year old son was offered school during lockdown but didn't take the offer as I don't want him to catch the virus - he is depressed at home’*

*‘Mental health of children (especially youngest). Desperately missing social interaction with friends, school and all his sporting activities. He is getting increasingly angry’*

***Home schooling.*** The open text questions revealed home schooling to be a source of tension within the home and parents were worried about their children becoming demotivated, getting behind at school, their future prospects if they were nearing important exams and poor behaviour. Some parents reported finding it particularly difficult to home school and keep their children entertained and focused if they had special educational needs like autism or ADHD. Problems with home schooling were compounded by a lack of necessary devices like laptops or tablets and issues with broadband capacity.

*‘Every day is a challenge to get our 11 year old daughter out of bed before lunchtime and engaged in an activity’*

*‘Worrying about our children's education and mental health. Both have autism and are finding the change in situation very challenging’*

*My son has behavioural problems partly due to medical reasons these behaviour issues have got a lot worse than normal due to all the change, no school, no routine, not seeing any family or friends’*

***Other worries and concerns.*** Many participants reported stress and anxiety around food shopping. There was a lot of fear around attending supermarkets and catching the virus, difficulties finding specific grocery items and catering for children with dietary requirements. Others shared their concerns about coming out of lockdown, being anxious about going into crowded places and what the new normal might look like.

‘ *How will I manage things after the lockdown, as it makes me anxious people not standing at a distance and I worry how I will gel into the situation when we are outside and getting back into normal’*

There were also a large number of participants concerned about what the virus and subsequent government measures would mean for wider society. They were worried about the country’s economic and political future and how people who were more vulnerable than themselves were coping. Participants reported feeling worried about the future, feeling helpless, unable to switch off from the news cycle and struggling to manage negative emotions around their children and offer them comfort.

*‘Trying to be strong for my children and convince them that everything is going to be okay’*

*‘How the 'new' normal is going to affect our lives moving forward. Work, sports clubs, socialising, seeing family etc.’*

*‘How and when will it be safe to go back to normality, how long is it going to last, will it get worse?’*

***Positive aspects of lockdown.*** One of the open-ended questions did enable people to report some positive aspects of the lockdown. When asked if there was anything that had become easier or more enjoyable since lockdown, some participants reported that nothing had improved, with many more reporting some positive consequences. These included getting to spend more quality time with their children, enjoying a slower pace of life, a more relaxed routine and spending less time driving and commuting. With less rushing to get to and from school, work and extracurricular activities, a number of participants reported that they were sleeping better, taking more time to cook meals and eating together as a family.

*‘Life has become a lot more relaxed over the last 3 weeks, no manic mornings trying to get everybody out of the house, time with kids, doing stuff with kids I would normally say 'not now' to. Get to know kids more. More time outside, [doing] jobs in the house that need doing’*

*‘Being together with children and family. There has been more family time as usually life is so busy and the children are at school or with their friends. Have enjoyed every minute of being together more as a family’*

Some participants who had children with health issues and/or special educational needs reported that it was easier to manage within the home:

*‘It's been easier to support my son and help him gain weight, he always eats better at home than at school so he has managed to gain a bit of weight’*

*‘Not having to get kids ready for school and also as my eldest has epilepsy he had constant seizures in school and at home he's not had one’*

## Discussion

This Covid-19 survey within an ongoing longitudinal study describes some of the key experiences of families living in the deprived and ethnically diverse city of Bradford during the first Covid-19 lockdown (March 23rd 2020). The findings have highlighted inequalities in living circumstances with a large proportion of ethnic minority families and those who are financially insecure having endured this stage of the pandemic in poor and overcrowded housing conditions. Economic insecurities were frequently reported with more than one-third reporting financial insecurity, and one-in-ten reporting serious economic difficulties such as having to regularly skip meals and serious concerns about being evicted or having their home repossessed. In addition, there were strong associations between financial insecurity and poor family relationships, mental health and negative health behaviours.

We found that many families who had lost income due to being self-employed and not able to work, being furloughed, or recently unemployed, found themselves in perilous financial, food and employment insecurity. Importantly, there was also higher financial insecurity reported in families who had needed to self isolate. Whilst the furlough scheme and support to self-employed workers was designed to provide support during this difficult time, our findings suggest that the loss of income for those on low wages is enough to tip families into financial difficulty, and potentially further exacerbate health inequalities. These findings reflect other research that has highlighted ethnic minority and deprived families as at high risk of financial and food insecurities ^5, 6^.

Another major concern uncovered in the survey was the mental health of mothers, with 18% reporting clinically significant depression symptoms and 16% clinically significant anxiety symptoms. Many participants also raised concerns about the mental health of their children. These findings reflect depression and anxiety prevalence rates reported in other lockdown surveys ^3, 4^. Depression was associated with White British ethnicity and both depression and anxiety were associated with financial insecurity, in addition to a number of other variables including poor housing, poor health and loneliness. Further exploration of this finding is planned to understand the underlying causes of increased poor mental health and ethnic differences.

Policy makers and commissioners must intervene to provide greater support to these families. Families need support to enable them to manage financially and stop them becoming homeless and living in food poverty. Increasing access to support for mental wellbeing is also critical during and after the pandemic. Whilst specialist services should focus on treating those with moderate to severe depression and anxiety, commissioners and policy makers must also consider broader, population-based preventative measures for those with mild symptoms.

Whilst the physical health of families was good overall, participants who smoked or drank alcohol often reported smoking/drinking more during lockdown and a large number reported doing less, or no exercise. Poor housing and lack of access to outdoor space were also common in this population. The increase of negative health behaviours in addition to living in poor housing conditions with a lack of access to safe green space puts people at risk of developing, or exacerbating, non-communicable diseases and co-morbidities such as diabetes, hypertension and respiratory illnesses, conditions that in turn increase risk of worse outcomes of the Covid-19 virus.

More than one quarter of families had a person vulnerable to Covid-19 living in their household. Our open ended questions identified that health anxieties about catching the Covid-19 virus and becoming severely ill or dying was the most commonly reported worry and had a negative impact on behaviors such as exercise. There has been concern across the UK about the dramatic drop in use of health services, and a lack of uptake of school places, especially for vulnerable children during lockdown. Given the high anxiety of being exposed to Covid-19, particularly in families living with a vulnerable person, methods to reassure and encourage vulnerable families to access critical services, return to work and school and engage in exercise needs to be considered extremely carefully and sensitively.

The free text responses complemented the findings from the quantitative survey, but also highlighted: high levels of worry and fear around the virus which had changed families behaviours and concerns around children’s mental health and the challenges of home schooling. Some families also noted positive aspects of the lockdown including less stress and better quality family time. These findings provide helpful insights for future research which we aim to focus on childrens’ mental health, childrens’ home learning / return to school, and what factors provide resilience in families to enable them to recover quickly from the pandemic.

### Strengths and limitations

These findings demonstrate a host of negative experiences during the first UK Covid-19 lockdown for families living in the ethnically diverse and deprived city of Bradford. We used multiple methods to obtain a high response in a timely way, but acknowledge that the overall low response may have introduced selection bias. Comparing results with other studies of similar and differing populations will be important to gain a fuller picture of the impact of the pandemic and its management on social and health inequalities. The longitudinal nature of our research with the BiB cohorts will allow us to look for change over time, comparing pre-pandemic data, as well as continued follow up of families over a one year period from April 2020 to March 2021 thereby adding more value to this research. Since this survey, the UK has experienced two further national lockdowns, as well as regional restrictions in between, and results reported here will form a baseline for understanding the ongoing and cumulative impact of the pandemic.

## Conclusion

The effect of the pandemic and lockdown may be socially patterned, with the most vulnerable in society bearing the brunt. Vulnerable families could be pushed into poverty and worsening mental ill health. There is a need for policy makers and commissioners to consider how to better support vulnerable families to enable them to manage financially and avoid them becoming homeless and living in debt and food poverty. There is also a need to provide support for a significant proportion of people who are now suffering from depression and anxiety, enabling services for severe cases and preventative interventions for those with mild symptoms to stop these getting worse. There is also a need to develop methods to reassure and encourage vulnerable families to access health and education services with immediate effect to stop these health inequalities becoming even worse.

## Data availability

### Underlying data

Scientists are encouraged and able to use BiB data, which are available through a system of managed open access. The steps below describe how to apply for access to BiB data.

- Before you contact BiB, please make sure you have read our Guidance for Collaborators. Our BiB executive review proposals on a monthly basis and we will endeavor to respond to your request as soon as possible. You can find out about the different datasets which are available here. If you are unsure if we have the data that you need please contact a member of the BiB team ( borninbradford@bthft.nhs.uk).
- Once you have formulated your request please complete the ‘Expression of Interest’ form available here and send to the BiB Programme Director ( rosie.mceachan@bthft.nhs.uk).
- If your request is approved we will ask you to sign a collaboration agreement and if your request involves biological samples we will ask you to complete a material transfer agreement.

### Extended data

Harvard Dataverse: Extended data for this paper: Experiences of lockdown during the Covid-19 pandemic: descriptive findings from a survey of families in the Born in Bradford study. https://doi.org/10.7910/DVN/BS28KA ^15^.

This project contains the following extended data:

- Supplementary Tables 1–12.doc
- COVID_BiBAndBiBBSFamilyQuestionnaire_v1.pdf

Data are available under the terms of the Creative Commons Zero "No rights reserved" data waiver (CC0 1.0 Public domain dedication).

## Footnotes

1 On the furlough scheme, eligible employees were paid 80% of their usual wages by the Government
