# Supplementary material for: Experiences of lockdown during the Covid-19 pandemic: descriptive findings from a survey of families in the Born in Bradford study
Source: Wellcome Open Res. 2021 Feb 26;5:228. Originally published 2020 Oct 2. [Version 2] doi: 10.12688/wellcomeopenres.16317.2 (PMC7927208; doi:10.12688/wellcomeopenres.16317.2)
Supplement: Peer review history

# Peer review: Reviewer response for version 2

**DOI:** 10.21956/wellcomeopenres.18345.r42860

**Published:** 2021-03-02

**Authors:** Sharon Goldfeld

**Affiliations:** 1 Policy and Equity, Murdoch Children's Research Institute, Melbourne, Australia; 2 Population Health, Murdoch Children's Research Institute, Melbourne, Australia

I have read and reviewed the article and the authors have addressed the concerns from both reviewers. There is far greater clarity and  the results are more aligned to the original research questions. The bar graphs are a very useful way of depicting the data.

Is the work clearly and accurately presented and does it cite the current literature?

Yes

If applicable, is the statistical analysis and its interpretation appropriate?

Yes

Are all the source data underlying the results available to ensure full reproducibility?

Yes

Is the study design appropriate and is the work technically sound?

Partly

Are the conclusions drawn adequately supported by the results?

Partly

Are sufficient details of methods and analysis provided to allow replication by others?

Yes

Reviewer Expertise:

Child public health, health inequities, public policy, paediatrics.

I confirm that I have read this submission and believe that I have an appropriate level of expertise to confirm that it is of an acceptable scientific standard.

# Peer review: Reviewer response for version 1

**DOI:** 10.21956/wellcomeopenres.17936.r40697

**Published:** 2020-11-30

**Authors:** Sharon Goldfeld

**Affiliations:** 1 Policy and Equity, Murdoch Children's Research Institute, Melbourne, Australia; 2 Population Health, Murdoch Children's Research Institute, Melbourne, Australia

Thank you for the opportunity to review this paper which was a pleasure to read. The results from this survey demonstrate firstly that, as we have always known, the social determinants continue to be the source of inequity for families and their children regardless of the pandemic. Both BBGU and BiBBS have a long history of exploring these issues. The authors have presented wide ranging data from a survey in these 2 existing cohorts in Bradford, UK. These cohorts are known for their ethnic diversity and general disadvantage. The cohorts are more or less concurrent but with different child ages. The authors have surveyed these families using a phone/postal survey in April-June 2020 which overlapped with the first wave and lockdown in response to COVID-19 in the UK. While the descriptive data are interesting, the volume of tables and lack of comparison to previous “states” means that it is difficult to both get a sense of the overall picture (too many tables) and to really understand the impact of the lockdown as opposed to the previous conditions and outcomes for these families. A potential reframe might be to focus on (1) where there are data to note the impact of the lockdown and pandemic using data that had questions focussed on “compared to” e.g. smoking or where there are data that are specific to the pandemic e.g. children learning from home or potentially those being furloughed and change in financial status and then (2) note that there is a substantial burden of disadvantage for these families that will inevitably worsen during the 2 waves of the pandemic (international data are already showing this) with COVID-19 exposing the inequity gaps in our systems.

 Further details are outlined below.

**Introduction:**

 While noting that the aim was to describe the various outcomes for these families, it may be more helpful to have a sense of what the authors might hypothesise here in terms of the differential impact of the lockdown.

**Methods**

 The descriptive methods here are quite straightforward.

**Result**

 It was difficult to get a clear view of the differential impact of the lock down itself on specific populations and /or the different cohorts. There are too many tables each of which are quite complex to read. I wonder if a number of them might go into a supplementary set of files but a key number related to the lockdown impact where those questions/measures exist could be highlighted in the paper itself. This might hook back into the actual research question being asked here i.e. is the question about the differential impact of lockdown/the pandemic on families.

 Given there are previous data I can only assume these comparisons will be published at a later time and hence the use of questions that I am sure align with previous cohort data collection. The strongest data related to those whose employment/financial status had changed. For this paper this would make the most sense to have as the anchor for all the tables and then to have the other tables in the supplementary files noting they might form the basis of another paper with comparative data and perhaps taking into account some of the confounding that might be contributing to the differences across ethnic groups. The qual data also aligns with the impact of loss of employment so works well.

 Of note the response rate of 28% was relatively low although it appears that the sample is relatively representative of the overall sample with some exceptions such as seemingly lower proportions of Pakistani families.

 The graphs were definitely easier to read than the tables

**Discussion/Conclusion:**

 I think the challenge of this paper is to anchor it more to a research question as it currently reads more like a report. The authors’ conclusions regard the importance of noting the vulnerability of populations even before COVID-19 is well placed. Data to support how COVID-19 has worsened people’s circumstances would strengthen this paper.

Is the work clearly and accurately presented and does it cite the current literature?

Yes

If applicable, is the statistical analysis and its interpretation appropriate?

Yes

Are all the source data underlying the results available to ensure full reproducibility?

Yes

Is the study design appropriate and is the work technically sound?

Partly

Are the conclusions drawn adequately supported by the results?

Partly

Are sufficient details of methods and analysis provided to allow replication by others?

Yes

Reviewer Expertise:

Child public health, health inequities, public policy, paediatrics.

I confirm that I have read this submission and believe that I have an appropriate level of expertise to confirm that it is of an acceptable scientific standard, however I have significant reservations, as outlined above.

# Response

**Published:** 2021-02-08

**Authors:** Josie Dickerson

**Affiliations:** Bradford Hospitals National Health Service Trust, UK

Thank you for your detailed review and helpful comments. We have submitted a revised version to re-frame the paper as suggested by this reviewer:

**​​​​​​​1.** **Introduction**

- We have added clarity to the introduction regarding our hypothesis – that health inequalities are likely to have worsened during the pandemic and we intend to look at differential impacts based on ethnicity and current financial insecurity.

- We have clarified the intentions of the wider programme of research in the introduction:
- a) That this paper reports the participant characteristics and the immediate health, social and economic status (by ethnicity and financial insecurity) during the first  survey of BiB parents undertaken during April – June 2020. One of our underlying aims of this paper was to make our Covid-19 survey findings widely available to researchers and policy makers as quickly as possible.
- b) That future plans are in place to use longitudinal data from before the pandemic, and from future surveys during the pandemic, to describe the trajectories, and identify the long-term consequences, of the pandemic on vulnerable populations.

**2. Results**

- The majority of the detailed tables are now supplementary tables, and we have used figures to more easily describe the data.

- As above, the results section is now hooked onto the hypothesis regarding differential impacts for ethnic minorities and those experiencing current financial insecurity. Other associations found are then described where relevant

**3. Discussion / Conclusion**

- We have added in references to recent papers that show similar findings to ours within England, further elaborated on the strengths / limitations section, and added in more about future research questions.

# Peer review: Reviewer response for version 1

**DOI:** 10.21956/wellcomeopenres.17936.r40699

**Published:** 2020-11-09

**Authors:** Malavika Subramanyam

**Affiliations:** 1 Social Epidemiology, Indian Institute of Technology Gandhinagar, Gandhinagar, India

This study describes the findings from a detailed survey of living conditions, psychological well-being, stresses, and lived experiences of a multi-ethnic socioeconomically disadvantaged sub-sample of the Born in Bradford cohorts during the Covid-19 lockdown. Unsurprisingly, the authors find that substantial proportions of the analytical sample lives in poor quality housing, has economic, housing, and food insecurities. Moreover, it was the socioeconomically disadvantaged sub-groups that reported greater proportions of depression and anxiety symptoms. The study underscores the burden of housing, economic, and psychological health issues in this population.

 An international audience would benefit from a brief comparison of the socioeconomic profile of the final analytical sample with those of Bradford and other major urban areas in England. A note on the unique contribution of the current study, beyond providing data to policy-makers interested specifically in Bradford, would also be helpful.

 The reader would also benefit from seeing a comparison of these lockdown prevalences of mental health outcomes with pre-pandemic prevalences in the same population. Similarly, it would be helpful to identify how the prevalence of the outcomes in this study compare with similar figures from the rest of England (or at least samples similar to the general English population).

 One stated goal of the study was the "identify any other areas of concern for future research questions". It would have been helpful if the authors had expanded more on this point. The free-text responses to the open-ended questions appear to have provided rich data. Perhaps those responses lead to interesting questions for the future?

 A few clarifications will further strengthen this excellent and detailed descriptive study. For instance, the abstract could clarify that a mix of telephonic, online, and paper-based surveys were used. And on a related note, a clarification could be added to the main text about any differences in the pattern of answers by the mode of survey data collection 

 Finally, the authors are requested to highlight a key finding which is specific and policy-relevant in the "conclusions" section of the abstract. For instance, perhaps they could offer a comparison with other cities/sub-groups or comment on the policies/programs in place which may need to be tweaked on the basis of these findings?

Is the work clearly and accurately presented and does it cite the current literature?

Yes

If applicable, is the statistical analysis and its interpretation appropriate?

Yes

Are all the source data underlying the results available to ensure full reproducibility?

Partly

Is the study design appropriate and is the work technically sound?

Yes

Are the conclusions drawn adequately supported by the results?

Yes

Are sufficient details of methods and analysis provided to allow replication by others?

Yes

Reviewer Expertise:

Social epidemiology.

I confirm that I have read this submission and believe that I have an appropriate level of expertise to confirm that it is of an acceptable scientific standard.

# Response

**Published:** 2021-02-08

**Authors:** Josie Dickerson

**Affiliations:** Bradford Hospitals National Health Service Trust, UK

Thank you for your review. We have noted the requests for clarity and trust that the revised version now provides that clarity, in particular:  

- Method of data collection have been added to the abstract
- The abstract conclusion has been amended to highlight how we will use the longitudinal data to make more generalizable findings relevant to national policy.
- The discussion highlights how the sample compares to other findings from England
- The discussion highlights the unique contribution that this longitudinal study makes, and that this is the first of a planned series of follow-ups and analyses using longitudinal data to further explore changes in mental health and economic insecurity from pre-pandemic to during the pandemic.

We have also expanded on the identification of future research questions, particularly from the free text responses in the discussion section. Thank you for noting this omission.

 We have not looked at differences in responses by completion mode (e.g. by phone/paper), but agree that this would be interesting. We plan to look at this, as well as potential differences in responses by time (e.g. immediate, compared to later lockdown) and will follow this up, perhaps as a separate methods paper.
